# Bis(2-methyl-4-nitro­anilinium) tetra­chloridomercurate(II)

**DOI:** 10.1107/S1600536808038415

**Published:** 2008-11-22

**Authors:** Jasrotia Dinesh, Melanie Rademeyer, David G. Billing, Andreas Lemmerer

**Affiliations:** a School of Chemistry, University of KwaZulu-Natal, Pietermaritzburg Campus, Private Bag X01, Scottsville 3209, South Africa; b Department of Chemistry, University of Pretoria, Pretoria 0002, South Africa; c Molecular Sciences Institute, School of Chemistry, University of the Witwatersrand, Private Bag 3, PO Wits 2050, South Africa

## Abstract

The title compound, (C_7_H_9_N_2_O_2_)_2_[HgCl_4_], self-assembles into cationic organic bilayers containing the 2-methyl-4-nitro­anilinium cations, sandwiched between anionic inorganic layers built up by the distorted tetra­hedral [HgCl_4_]^2−^ groups. The organic sheets are inter­linked through weak C—H⋯O hydrogen bonds, while they inter­act with the anionic part *via* strong charge-assisted N^+^—H⋯Cl—Hg hydrogen bonds. The [HgCl_4_]^2−^ anions are bis­ected by a mirror plane passing through the metal and two of the chloride ions.

## Related literature

The structures of bis­(2-methyl-4-nitro­anilinium) tetra­chloro­cadmate (Azumi *et al.*, 1996 ▶) as well as those of the bromide and iodide salts of 2-methyl-4-nitro­anilinium (Lemmerer & Billing, 2006 ▶) have already been reported. For related literature on C—H⋯O_nitro_ inter­actions, see: Sharma & Desiraju (1994 ▶). 
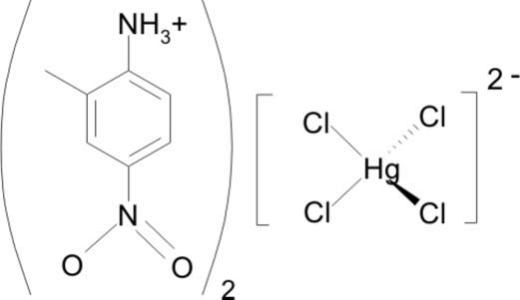


## Experimental

### 

#### Crystal data

(C_7_H_9_N_2_O_2_)_2_[HgCl_4_]
*M* *_r_* = 648.71
Orthorhombic, 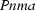

*a* = 8.2527 (11) Å
*b* = 30.059 (4) Å
*c* = 8.3038 (10) Å
*V* = 2059.9 (5) Å^3^
*Z* = 4
Mo *K*α radiation
μ = 8.02 mm^−1^
*T* = 173 (2) K
0.42 × 0.25 × 0.16 mm

#### Data collection

Bruker SMART CCD area-detector diffractometer
Absorption correction: integration (*XPREP*; Bruker, 1999 ▶) *T*_min_ = 0.609, *T*_max_ = 0.757
10307 measured reflections
3174 independent reflections
2197 reflections with *I* > 2σ(*I*)
*R*_int_ = 0.091

#### Refinement

*R*[*F* ^2^ > 2σ(*F* ^2^)] = 0.053
*wR*(*F* ^2^) = 0.152
*S* = 1.09
3174 reflections
129 parameters
H-atom parameters constrained
Δρ_max_ = 1.05 e Å^−3^
Δρ_min_ = −2.89 e Å^−3^

### 

Data collection: *SMART-NT* (Bruker, 1998 ▶); cell refinement: *SAINT-Plus* (Bruker, 1999 ▶); data reduction: *SAINT-Plus*; program(s) used to solve structure: *SHELXS97* (Sheldrick, 2008 ▶); program(s) used to refine structure: *SHELXL97* (Sheldrick, 2008 ▶); molecular graphics: *DIAMOND* (Brandenburg, 2006 ▶) and *Mercury* (Bruno *et al.*, 2002 ▶); software used to prepare material for publication: *WinGX* (Farrugia, 1999 ▶) and *PLATON* (Spek, 2003 ▶).

## Supplementary Material

Crystal structure: contains datablocks global, I. DOI: 10.1107/S1600536808038415/bg2223sup1.cif

Structure factors: contains datablocks I. DOI: 10.1107/S1600536808038415/bg2223Isup2.hkl

Additional supplementary materials:  crystallographic information; 3D view; checkCIF report

## Figures and Tables

**Table 1:** Hydrogen-bond geometry (Å, °)

*D*—H⋯*A*	*D*—H	H⋯*A*	*D*⋯*A*	*D*—H⋯*A*
N1—H6⋯Cl2	0.91	2.76	3.257 (6)	116
N1—H4⋯Cl1^i^	0.91	2.35	3.248 (6)	170
N1—H5⋯Cl1^ii^	0.91	2.49	3.381 (7)	167
N1—H6⋯Cl3^iii^	0.91	2.54	3.241 (7)	134
C3—H1⋯O1^iv^	0.95	2.52	3.424 (10)	160

Symmetry codes: (i) 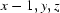; (ii) 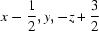; (iii) 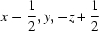; (iv) 
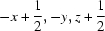
.

**Fig. 1.**
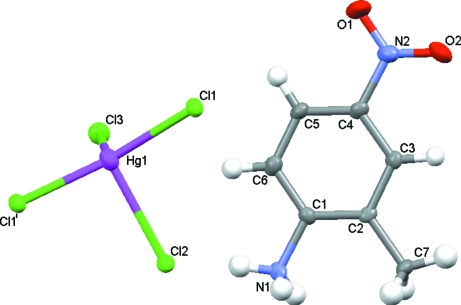
Atom labelling scheme of (I) with thermal ellipsoids drawn at the 50% probability level. The Cl atom marked with a prime (') is at symmetry position (1/2 + x, 1/2 - y, 1/2 - z).

**Fig. 2.**
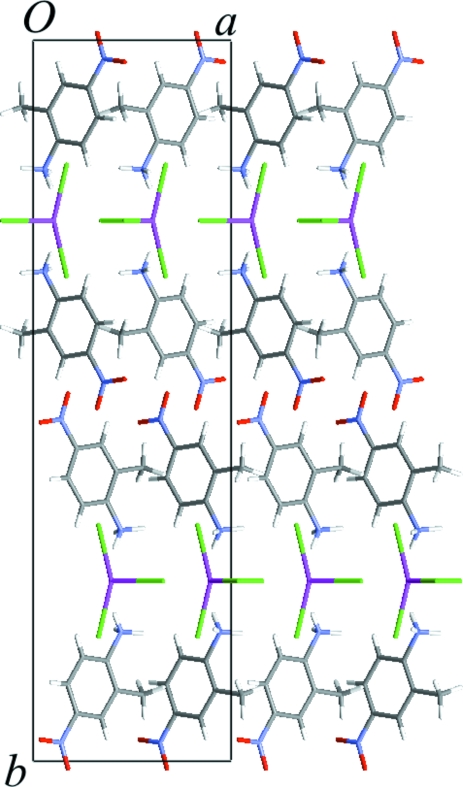
Packing arrangement viewed down the c-axis.

## supplementary crystallographic information

### Comment

As part of a study focused on the fundamental understanding of the non-covalent interactions occurring in organic-inorganic hybrids, the structure of bis(2-methyl-4-nitroanilinium) tetrachloromercurate, 2(C_7_H_9_N_2_O_2_)^+^.(HgCl_4_)^2-^, (I), was determined. It was found that the title compound is isostructural to the previously reported hybrid, bis(2-methyl-4-nitroanilinium) tetrachlorocadmate (Azumi *et al.*, 1996). The structures of the bromide and iodide salts of the 2-methyl-4-nitroanilinium cation have already been reported (Lemmerer & Billing, 2006).

The molecular geometry and atomic numbering scheme of (I) are illustrated in Fig. 1. The asymmetric unit contains one 2-methyl-4-nitroanilinium cation and a HgCl_4_^2-^ anion, halved by a mirror plane (x, 1/2-y, z) passing through the metal and two of the chlorine ions. The structure consists of alternating, non-interdigitated organic bilayers containing the 2-methyl-4-nitroanilium cations, and inorganic layers containing the isolated (HgCl_4_)^2-^ anions (Fig. 2.).

In the organic bilayers the nitro groups pack in the centre of the layer, in a tail-to-tail arrangement, and the aromatic ring plane (C1->C6) forms an angle of 86.3° to the inorganic layer plane. It has been reported by Sharma and Desiraju (1994) that weak C—H···O interactions, with the nitro group as a hydrogen bond acceptor occurs in many unsaturated compounds, despite the fact that the nitro group is not very basic, and it is precisely this type of interaction the one which links both organic layers in (I): atom C3 on the aromatic ring at symmetry position (1/2 - *x*, 1/2 + *y*, *z* - 1/2) acts as proton donor while the O1 of nitro group at symmetry position (*x*, 1/2 - *y*, *z*) acts as acceptor, with an H···O distance of 2.52 Å.

The organic and inorganic layers are linked through charge assisted N^+^—H···Cl—Hg hydrogen bonds, with the hydrogen bonding interactions listed in Table 1. N1 is the only hydrogen bond donor with all three hydrogen atoms involved in hydrogen bonding. Atom H6 is shared by two chlorine atoms (Cl2 at symmetry position: (*x*, *y*, *z*) and Cl3 at symmetry position: (*x* - 1/2, 1/2 - *y*, 1/2 - *z*)) and thus forms a bifurcated interaction. Two approximately linear hydrogen bonds are formed through atoms H4 and H5 with Cl1 at symmetry positions (*x* - 1, *y*, *z*) and (*x* - 1/2, *y*, 1/2 - *z*), respectively. All four chloro ligands on the HgCl_4_^2-^ anion act as hydrogen bond acceptors.

### Experimental

Compound (I) was prepared by the addition of 0.097 g (0.357 mmol) of HgCl_2_ (Aldrich) and 0.102 g (0.333 mmol) of 2-methyl-4-nitroaniline (Aldrich) to 6 ml of 33% HCl. Complete dissolution was obtained after refluxing at 90°C for 12 h in an oil bath. Slow cooling in oil bath over 48 h produced the crystals. A colourless crystal of 0.42 *x* 1/4*x* 0.16 mm was used for X-ray data collection.

### Refinement

H atoms were placed geometrically and refined in idealized positions in the riding-model approximation, with C—H 0.95 (ArH) and 0.98 Å (CH_3_) and N—H = 0.91 Å; *U*_iso_(H) = 1.5*U*_eq_(N), 1.5*U*_eq_(C) for methyl H atoms and 1.2*U*_eq_(C) for other H atoms. The highest residual peaks in the final ΔF syntheses lie at 0.90 Å from Cl3.

### Crystal data

**Table:**

(C_7_H_9_N_2_O_2_)_2_[HgCl_4_]	*D*_x_ = 2.092 Mg m^−^^3^
*M**_r_* = 648.71	Melting point: 441 K
Orthorhombic, *P**n**m**a*	Mo *K*α radiation λ = 0.71073 Å
Hall symbol: -P 2ac 2n	Cell parameters from 937 reflections
*a* = 8.2527 (11) Å	θ = 3.2–28.3º
*b* = 30.059 (4) Å	µ = 8.02 mm^−^^1^
*c* = 8.3038 (10) Å	*T* = 173 (2) K
*V* = 2059.9 (5) Å^3^	Plate, colourless
*Z* = 4	0.42 × 0.25 × 0.16 mm
*F*_000_ = 1240	

### Data collection

**Table:**

Bruker SMART CCD area-detector diffractometer	3174 independent reflections
Radiation source: fine-focus sealed tube	2197 reflections with *I* > 2σ(*I*)
Monochromator: graphite	*R*_int_ = 0.091
*T* = 173(2) K	θ_max_ = 32.2º
ω scans	θ_min_ = 1.4º
Absorption correction: integration(XPREP; Bruker, 1999)	*h* = −11→12
*T*_min_ = 0.609, *T*_max_ = 0.757	*k* = −38→44
10307 measured reflections	*l* = −8→11

### Refinement

**Table:**

Refinement on *F*^2^	Secondary atom site location: difference Fourier map
Least-squares matrix: full	Hydrogen site location: inferred from neighbouring sites
*R*[*F*^2^ > 2σ(*F*^2^)] = 0.053	H-atom parameters constrained
*wR*(*F*^2^) = 0.152	*w* = 1/[σ^2^(*F*_o_^2^) + (0.077*P*)^2^ + 1.1955*P*] where *P* = (*F*_o_^2^ + 2*F*_c_^2^)/3
*S* = 1.09	(Δ/σ)_max_ = 0.003
3174 reflections	Δρ_max_ = 1.05 e Å^−^^3^
129 parameters	Δρ_min_ = −2.89 e Å^−^^3^
Primary atom site location: structure-invariant direct methods	Extinction correction: none

### Special details

**Table:**

Experimental. Numerical integration absorption corrections based on indexed crystal faces were applied using the *XPREP* routine (Bruker, 2004)
Geometry. All e.s.d.'s (except the e.s.d. in the dihedral angle between two l.s. planes) are estimated using the full covariance matrix. The cell e.s.d.'s are taken into account individually in the estimation of e.s.d.'s in distances, angles and torsion angles; correlations between e.s.d.'s in cell parameters are only used when they are defined by crystal symmetry. An approximate (isotropic) treatment of cell e.s.d.'s is used for estimating e.s.d.'s involving l.s. planes.
Refinement. Refinement of *F*^2^ against ALL reflections. The weighted *R*-factor *wR* and goodness of fit *S* are based on *F*^2^, conventional *R*-factors *R* are based on *F*, with *F* set to zero for negative *F*^2^. The threshold expression of *F*^2^ > σ(*F*^2^) is used only for calculating *R*-factors(gt) *etc*. and is not relevant to the choice of reflections for refinement. *R*-factors based on *F*^2^ are statistically about twice as large as those based on *F*, and *R*- factors based on ALL data will be even larger.

### Fractional atomic coordinates and isotropic or equivalent isotropic displacement parameters (Å^2^)

**Table:**

	*x*	*y*	*z*	*U*_iso_*/*U*_eq_	
Hg1	0.60393 (5)	0.2500	0.46538 (6)	0.02730 (16)	
Cl3	0.4637 (3)	0.2500	0.1888 (3)	0.0232 (5)	
Cl1	0.6690 (2)	0.17293 (6)	0.5175 (2)	0.0198 (3)	
C1	0.1288 (8)	0.1395 (2)	0.5286 (9)	0.0167 (13)	
C6	0.2495 (9)	0.1443 (3)	0.4129 (9)	0.0210 (15)	
H3	0.2794	0.1731	0.3760	0.025*	
O1	0.4612 (9)	0.0300 (2)	0.2467 (9)	0.0504 (19)	
O2	0.3238 (8)	−0.00979 (19)	0.4124 (8)	0.0394 (15)	
N1	0.0546 (7)	0.1794 (2)	0.5922 (8)	0.0194 (12)	
H6	0.0891	0.2034	0.5347	0.029*	
H5	0.0832	0.1828	0.6973	0.029*	
H4	−0.0551	0.1772	0.5845	0.029*	
C2	0.0779 (7)	0.0982 (2)	0.5872 (8)	0.0151 (13)	
N2	0.3592 (7)	0.0258 (2)	0.3538 (8)	0.0252 (14)	
C4	0.2790 (8)	0.0663 (3)	0.4125 (8)	0.0189 (14)	
C3	0.1558 (8)	0.0608 (2)	0.5265 (8)	0.0171 (13)	
H1	0.1257	0.0319	0.5619	0.021*	
C5	0.3261 (8)	0.1068 (2)	0.3516 (8)	0.0199 (14)	
H2	0.4075	0.1091	0.2711	0.024*	
C7	−0.0544 (9)	0.0924 (2)	0.7085 (9)	0.0223 (16)	
H9	−0.0626	0.0610	0.7383	0.033*	
H8	−0.1575	0.1023	0.6621	0.033*	
H7	−0.0299	0.1102	0.8045	0.033*	
Cl2	0.3444 (3)	0.2500	0.6670 (3)	0.0199 (5)	

### Atomic displacement parameters (Å^2^)

**Table:**

	*U*^11^	*U*^22^	*U*^33^	*U*^12^	*U*^13^	*U*^23^
Hg1	0.0319 (2)	0.0206 (2)	0.0293 (3)	0.000	0.00001 (19)	0.000
Cl3	0.0272 (13)	0.0262 (13)	0.0162 (11)	0.000	−0.0057 (10)	0.000
Cl1	0.0186 (8)	0.0166 (8)	0.0243 (8)	0.0002 (6)	−0.0007 (7)	0.0008 (7)
C1	0.018 (3)	0.016 (3)	0.017 (3)	0.000 (3)	−0.003 (3)	−0.002 (3)
C6	0.022 (3)	0.024 (4)	0.017 (3)	−0.006 (3)	0.000 (3)	−0.001 (3)
O1	0.057 (4)	0.039 (4)	0.055 (4)	−0.007 (4)	0.038 (4)	−0.013 (3)
O2	0.047 (4)	0.020 (3)	0.051 (4)	0.005 (3)	0.012 (3)	−0.007 (3)
N1	0.010 (2)	0.018 (3)	0.030 (3)	−0.001 (2)	−0.003 (2)	0.000 (3)
C2	0.010 (3)	0.023 (4)	0.013 (3)	0.000 (2)	−0.003 (2)	0.001 (3)
N2	0.020 (3)	0.027 (4)	0.030 (4)	0.000 (3)	0.004 (2)	−0.007 (3)
C4	0.019 (3)	0.025 (4)	0.013 (3)	−0.003 (3)	0.001 (3)	−0.004 (3)
C3	0.015 (3)	0.019 (3)	0.017 (3)	−0.004 (3)	−0.003 (3)	−0.002 (3)
C5	0.018 (3)	0.024 (4)	0.018 (3)	−0.004 (3)	0.005 (3)	−0.001 (3)
C7	0.021 (3)	0.022 (4)	0.024 (4)	0.001 (3)	0.010 (3)	0.003 (3)
Cl2	0.0143 (10)	0.0189 (11)	0.0266 (13)	0.000	0.0004 (9)	0.000

### Geometric parameters (Å, °)

**Table:**

Hg1—Cl1	2.4170 (18)	N1—H5	0.9100
Hg1—Cl1^i^	2.4170 (18)	N1—H4	0.9100
Hg1—Cl3	2.572 (2)	C2—C3	1.390 (10)
Hg1—Cl2	2.718 (2)	C2—C7	1.496 (10)
C1—C6	1.392 (10)	N2—C4	1.469 (10)
C1—C2	1.396 (10)	C4—C5	1.375 (10)
C1—N1	1.447 (9)	C4—C3	1.399 (10)
C6—C5	1.388 (10)	C3—H1	0.9500
C6—H3	0.9500	C5—H2	0.9500
O1—N2	1.230 (9)	C7—H9	0.9800
O2—N2	1.212 (9)	C7—H8	0.9800
N1—H6	0.9100	C7—H7	0.9800
			
Cl1—Hg1—Cl1^i^	146.85 (9)	C1—C2—C7	123.9 (6)
Cl1—Hg1—Cl3	105.06 (4)	O2—N2—O1	123.0 (7)
Cl1^i^—Hg1—Cl3	105.06 (4)	O2—N2—C4	119.3 (6)
Cl1—Hg1—Cl2	93.71 (5)	O1—N2—C4	117.6 (7)
Cl1^i^—Hg1—Cl2	93.71 (5)	C5—C4—C3	124.0 (7)
Cl3—Hg1—Cl2	101.28 (8)	C5—C4—N2	119.0 (6)
C6—C1—C2	123.2 (7)	C3—C4—N2	117.0 (6)
C6—C1—N1	117.9 (6)	C2—C3—C4	119.0 (7)
C2—C1—N1	118.9 (6)	C2—C3—H1	120.5
C5—C6—C1	119.7 (7)	C4—C3—H1	120.5
C5—C6—H3	120.2	C4—C5—C6	117.1 (6)
C1—C6—H3	120.2	C4—C5—H2	121.5
C1—N1—H6	109.5	C6—C5—H2	121.5
C1—N1—H5	109.5	C2—C7—H9	109.5
H6—N1—H5	109.5	C2—C7—H8	109.5
C1—N1—H4	109.5	H9—C7—H8	109.5
H6—N1—H4	109.5	C2—C7—H7	109.5
H5—N1—H4	109.5	H9—C7—H7	109.5
C3—C2—C1	117.0 (6)	H8—C7—H7	109.5
C3—C2—C7	119.1 (7)		
			
C2—C1—C6—C5	−0.6 (11)	O1—N2—C4—C3	175.8 (7)
N1—C1—C6—C5	178.6 (6)	C1—C2—C3—C4	0.2 (9)
C6—C1—C2—C3	1.0 (10)	C7—C2—C3—C4	179.7 (7)
N1—C1—C2—C3	−178.1 (6)	C5—C4—C3—C2	−2.1 (11)
C6—C1—C2—C7	−178.4 (7)	N2—C4—C3—C2	179.1 (6)
N1—C1—C2—C7	2.4 (10)	C3—C4—C5—C6	2.5 (11)
O2—N2—C4—C5	176.5 (7)	N2—C4—C5—C6	−178.7 (6)
O1—N2—C4—C5	−3.1 (10)	C1—C6—C5—C4	−1.2 (10)
O2—N2—C4—C3	−4.6 (10)		

Symmetry codes: (i) *x*, −*y*+1/2, *z*.

### Hydrogen-bond geometry (Å, °)

**Table:**

*D*—H···*A*	*D*—H	H···*A*	*D*···*A*	*D*—H···*A*
N1—H6···Cl2	0.91	2.76	3.257 (6)	116
N1—H4···Cl1^ii^	0.91	2.35	3.248 (6)	170
N1—H5···Cl1^iii^	0.91	2.49	3.381 (7)	167
N1—H6···Cl3^iv^	0.91	2.54	3.241 (7)	134
C3—H1···O1^v^	0.95	2.52	3.424 (10)	160

Symmetry codes: (ii) *x*−1, *y*, *z*; (iii) *x*−1/2, *y*, −*z*+3/2; (iv) *x*−1/2, *y*, −*z*+1/2; (v) −*x*+1/2, −*y*, *z*+1/2.
